# Supplementary material for: Top-Down Machine Learning of Coarse-Grained Protein Force Fields
Source: J Chem Theory Comput. 2023 Oct 24;19(21):7518–26. doi: 10.1021/acs.jctc.3c00638 (PMC10777392; doi:10.1021/acs.jctc.3c00638)
Supplement: Supplementary file 1 — ct3c00638_si_001.pdf [file ct3c00638_si_001.pdf]

Supporting Information
Top-down machine learning of coarse-grained protein force-fields

Carles Navarro,[†] Maciej Majewski,[†] and Gianni De Fabritiis[*,‡,¶,§]

†*Acellera Labs, Doctor Trueta 183, 08005, Barcelona, Spain*

‡*Computational Science Laboratory, Universitat Pompeu Fabra, Barcelona Biomedical Research Park (PRBB), Carrer Dr. Aiguader 88, 08003, Barcelona, Spain*

¶*Acellera Ltd, Devonshire House 582, HA7 1JS, United Kingdom*

§*Institució Catalana de Recerca i Estudis Avançats (ICREA), Passeig Lluis Companys 23, 08010 Barcelona, Spain*

E-mail: gianni.defabritiis@upf.edu

Prior energy terms

The pairwise bonded term was:

$$U_\lambda^{harmonic}(r) = k(r - r_0)^2 + V_0 \tag{1}$$

where r is the distance between beads of a given bond, r_0 is the equilibrium distance and k is the spring constant.

The nonbonded repulsive term was:

$$U_\lambda^{repulsive}(r) = 4\epsilon r^{-6} + V_0 \tag{2}$$

where ϵ is a constant fitted to the data and r is the distance between two beads. Finally, the dihedral prior was:

$$U_{\lambda}^{dihedral}(\phi) = \sum_{n=1,2} k_n(1 + cos(n\phi - \gamma_n)) \quad (3)$$

where ϕ is the dihedral angle between four consecutive beads, k_n is the amplitude and γ_n is the phase offset of the harmonic component of periodicity n. The parameters for the priors were fitted to data of all-atom simulations of the fast-folding proteins. For the dihedral prior we used specific dihedrals for each combination of four beads, and for the general NNP we considered all the four beads combinations equally since the straining dataset was bigger. In both cases, the values of k_n were divided by five to achieve a soft prior that does not disturb the simulation too much.

Graph Neural Networks

The Graph Neural Network used as an NNP is the same as the one in the publicly available package TorchMD-NET[2]. The network is inspired by SchNet[1] and PhysNet[3], and optimized for coarse-graining. The network takes as input the Cartesian coordinates of the coarse-grained beads, alongside a predetermined type for each bead. Each bead represents a node of the graph and is given an embedding feature vector by applying a learnable linear mapping. The series of network operations can be written as:

$$
\begin{aligned}
\xi^0 &= W^E z \\
\xi^1 &= \xi^0 + W^0 \sigma(Aggr(W^C * \xi^0)) \\
\xi^2 &= \xi^1 + W^1 \sigma(Aggr(W^C * \xi^1)) \\
&\vdots \\
\xi^N &= \xi^{N-1} + W^{N-1} \sigma(Aggr(W^C * \xi^{N-1})) \\
U &= H_{out}(\xi^N) \\
F &= -\operatorname{grad}(U, x)
\end{aligned}
$$

for N interaction layers. Where W^C are continuous filters, generated by expanding the pairwise distance between beads into a set of radial basis functions. $Aggr$ is an aggregation function that reduces the convolution output, in our case we choose the sum as the aggregation method. Finally, the graph level feature U is computed which, in our case, corresponds to the potential energy of the protein and that can be used to compute the forces acting on each bead with an autograd call with respect to the Cartesian coordinates.

Training curves

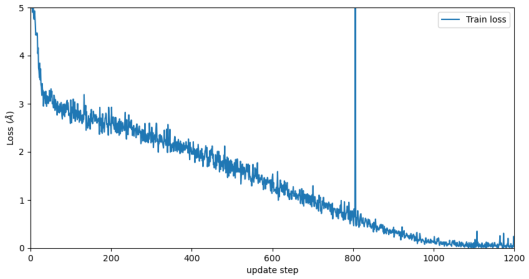

Figure S1: Train curve for transferable fast-folding potential.

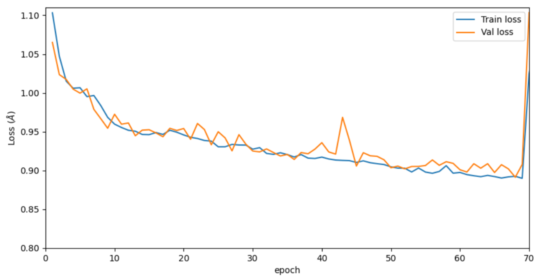

Figure S2: Train curve for the general NNP potential.

Time-lagged independent component analysis

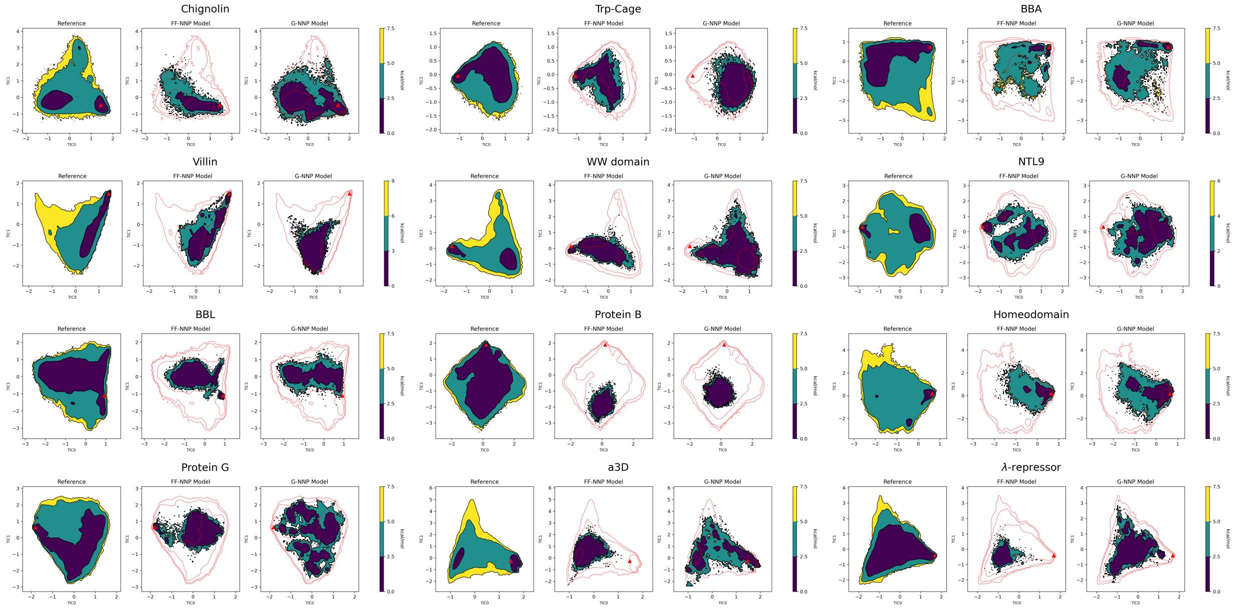

Figure S3: Comparative analysis of the free energy landscape derived from reference all-atom molecular dynamics (MD) (left), FF-NNP model (center), and G-NNP model (right) during coarse-grained simulations across the initial two TICA dimensions for each protein under study. The computation of the free energy landscape for each simulation group involved the segmentation of the first two TICA dimensions into a grid of 80x80 cells, followed by the calculation of the average weights of the equilibrium probability within each cell, as delineated by the Markov state model. The delineated red line marks the energy level above the free energy minimum indicated by the all-atom equilibrium density, characterized by values of 9kcal/mol for Villin and a3d, 6 kcal/mol for NTL9, and 7.5 kcal/mol for the remaining proteins.

Table S1: Fast-folding proteins length and sequence.

Protein	Length	Sequence
Chignolin	10	YYDPETGTWY
Trp-cage	20	DAYAQWLKDGGPSSGRPPPS
BBA	28	EQYTAKYKGRTFRNEKELRDFIEKFKGR
WW-domain	34	KLPPGWEKRMSRSSGRVYYFNHITNASQWERPSG
Villin	35	LSDEDFKAVFGMTRSAFANLPLWXQQHLXKEKGLF
NTL9	39	MKVIFLKDVKGMGKKGEIKNVADGYANNFLFKQGLAIEA
BBL	47	GSQNNDALSPAIRRLLAEWNLDASAIKGTGVGGRLTREDVEKHLAKA
Protein B	47	LKNAIEDAIAELKKAGITSDFYFNAINKAKTVEEVNALVNEILKAHA
Homeodomain	54	RPRTAFSSEQLARLKREFNENRYLTERRRQQLSSELGLNEAQIKIWFQNKRAKI
Protein G	56	DTYKLVIVLNGTTFTYTTEAVDAATAEKVFKQYANDAGVDGEWTYDAATKTFTVTE
a3D	73	MGSWAEFKQRLAAIKTRLQALGGSEAELAAFEKEIAAFESELQAYKGKGNPEVEALRKEAAAIRDELQAYRHN
λ-repressor	80	PLTQEQLEDARRLKAIYEKKKNELGLSQESVADKMGMGQSGVGALFNGINALNAYNAALLAKILKVSVEEFSPSIAREIY

Training hyperparameters

Different architecture hyperparameters were used for each dataset (Table S2). For the smaller dataset, we used a network with a small cutoff and less number of layers in order to have fewer parameters, which helped to avoid overfitting. 4 NVIDIA GeForce RTX 2080 machines were used for training the general NNP and 1 NVIDIA GeForce RTX 2080 for the fast-folders dataset.

Table S2: Hyperparameters choices for the neural networks trained on the fast folders and the monomers datasets. The neural network was implemented with the TorchMD-Net package[2], and the rest of the parameters were left to the default values.

Hyperparameter	Value for the Fast-Folders NNP	Value for the General NNP
Number of interaction layers	1	4
Activation function	tanh	tanh
Radial base function (RBF) type	expnorm	expnorm
Number of RBF	18	18
Upper cutoff for RBF	9.0	12.0
Lower cutoff for RBF	3.0	3.0
Trainable RBF	True	True
Model type	graph-network	graph-network
Embedding dimension	256	256
Learning rate	1.0e-4	5.0e-4
Neighbor embedding	False	False
Batch size	12	32
Total parameters	326437	1129765

Simulation speed

Simulations were performed using single NVIDIA GeForce GTX 1080 for the F-NNP and NVIDIA GeForce RTX 4090 for the G-NNP, in Table S3 we summarize the speed of the two models for each simulated system.

Table S3: Comparison of MD simulation speed (ns/day) of the FF-NNP and G-NNP. The results obtained with NVIDIA GTX 1080 for the FF-NNP and with NVIDIA GeForce RTX 4090 for the G-NNP.

Protein	Fast-Folders NNP (ns/day)	General NNP (ns/day)
Chignolin	21.48	50.56
Trp-cage	20.69	52.66
BBA	20.56	50.24
WW-domain	21.11	49.98
Villin	21.24	51.89
NTL9	21.40	50.20
BBL	20.62	48.89
Protein B	21.00	47.66
Homeodomain	11.52	49.23
Protein G	9.97	47.60
a3D	9.32	45.70
λ-repressor	8.70	44.08

References

[1] K. T. Schütt et al. "SchNet – A deep learning architecture for molecules and materials". In: *The Journal of Chemical Physics* 148.24 (June 2018), p. 241722.

[2] Philipp Thölke and Gianni De Fabritiis. "TorchMD-Net: Equivariant Transformers for Neural Network Based Molecular Potentials". In: *International Conference on Learning Representations (ICLR)*. Virtual Conference, 2022.

[3] Oliver T. Unke and Markus Meuwly. "PhysNet: A Neural Network for Predicting Energies, Forces, Dipole Moments, and Partial Charges". In: *Journal of Chemical Theory and Computation* 15.6 (2019). PMID: 31042390, pp. 3678–3693. DOI: 10.1021/acs.jctc.9b00181.
